# Long Noncoding RNA Mediated Regulation in Human Embryogenesis, Pluripotency, and Reproduction

**DOI:** 10.1155/2022/8051717

**Published:** 2022-01-22

**Authors:** Lei Liu, Fang Fang

**Affiliations:** The First Affiliated Hospital of USTC, Division of Life Sciences and Medicine, University of Science and Technology of China, Hefei, Anhui, China 230001

## Abstract

Long noncoding RNAs (lncRNAs), a class of noncoding RNAs with more than 200 bp in length, are produced by pervasive transcription in mammalian genomes and regulate gene expression through various action mechanisms. Accumulating data indicate that lncRNAs mediate essential biological functions in human development, including early embryogenesis, induction of pluripotency, and germ cell development. Comprehensive analysis of sequencing data highlights that lncRNAs are expressed in a stage-specific and human/primate-specific pattern during early human development. They contribute to cell fate determination through interacting with almost all classes of cellular biomolecules, including proteins, DNA, mRNAs, and microRNAs. Furthermore, the expression of a few of lncRNAs is highly associated with the pathogenesis and progression of many reproductive diseases, suggesting that they could serve as candidate biomarkers for diagnosis or novel targets for treatment. Here, we review research on lncRNAs and their roles in embryogenesis, pluripotency, and reproduction. We aim to identify the underlying molecular mechanisms essential for human development and provide novel insight into the causes and treatments of human reproductive diseases.

## 1. Introduction

Identification and functional characterization of noncoding RNAs (ncRNAs) have revolutionized our traditional view of RNA biology, as well as developmental biology [1]. Before discovering microRNAs (miRNAs) and small interfering RNAs, mRNAs that are transcribed from the coding region of the genome and translated as proteins are considered the primary regulators of the gene expression program in the cells [2]. The vast majority of the genome that is not translated into protein is junk DNA regions [3]. With the rapid development of microarray and high-throughput sequencing technology, a comprehensive annotation of the mammalian genome demonstrates that most mammalian genome is actively transcribed into RNAs, and thousands of ncRNAs have been identified [4, 5]. ncRNAs are divided into two main types according to the length of the transcripts: small noncoding RNAs (sncRNAs), which are composed of less than 200 nucleotides, and long noncoding RNAs (lncRNAs), which consist of more than 200 nucleotides [6]. In this review, we focused on the discussion of lncRNAs. There are five different sources of lncRNAs: (1) a protein-coding gene was mutated and transformed into a noncoding RNA sequence. (2) Following chromosome rearrangement, two separate nontranscribed sequence regions are juxtaposed together to produce expressed noncoding sequences. (3) lncRNAs without a protein-coding function are produced by duplicating noncoding genes by retrotransposition. (4) Local two tandem duplication produces adjacent repeat sequences, which increases the size of lncRNs. (5) The insertion of transposable elements (TEs) can produce functional lncRNAs [7, 8].

It was questionable whether lncRNAs have putative functions in cells, as they are present in relatively low levels. It is estimated that total lncRNAs are present at two magnitudes less than total mRNAs. However, recent research suggests that lncRNAs may function at a very low level as a molecular scaffold or a catalytic molecule [9]. A growing number of lncRNAs are found to play essential roles in regulating cell proliferation, survival, cell cycle, differentiation, and apoptosis [10]. They are also indicated as vital regulators in initiating and developing many diseases, including reproductive diseases [11]. X-inactive specific transcript (XIST), located on the X-chromosome of mammalian cells, is the first reported lncRNA. It has been proven to be a major regulator of the X-inactivation process [12]. Another well-established example of functional lncRNAs is H19, which is highly expressed in many tissues derived from endoderm and mesoderm. It regulates the network of imprinted genes that regulate fetal and postnatal growth [13], and it is differentially expressed in many disease tissues.

**Figure 1:**
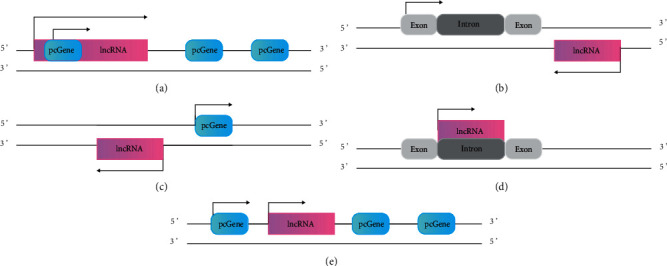
Schematic diagram of lncRNA classification. Classification of lncRNAs into five classes: (a) sense, (b) antisense, (c) bidirectional, (d) intronic, and (e) intergenic.

lncRNAs can be divided into five categories based on their genome localization and the direction of transcription relative to the protein-coding genes (pcGenes) in the genome: sense, antisense, bidirectional, intronic, and long intergenic (Figure 1) [14]. Sense lncRNAs are transcribed from the same strand and direction as pcGenes, and antisense lncRNAs are transcribed from the opposite strand of pcGenes. Sense and antisense lncRNAs are located within the regions of their surrounding pcGenes. Bidirectional lncRNA is located less than 1 kb from the surrounding pcGenes, sharing the same promoter as the protein-coding gene, but transcribed from the opposite direction [15]. Long intergenic noncoding RNAs (lincRNAs) are located within the intergenic regions of pcGenes, and they do not overlap with protein-coding regions.

**Figure 2:**
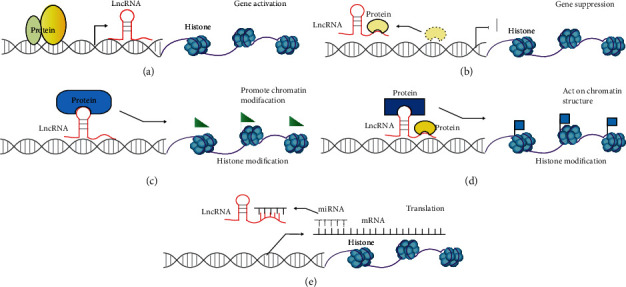
Schematic diagram of lncRNA mechanisms of action. Mechanisms of action: (a) signaling, (b) decoy, (c) guides, (d) scaffold, and (e) miRNA sponge.

lncRNAs could control transcription in *cis* or *trans*, regulate essential proteins or nucleic acid molecules, and are also involved in the organization of the nuclear domains [16]. The mechanisms of action vary depending on their structural conformations, biochemical properties, and specific subcellular localization [17, 18] (Figure 2). (1) They could function as signal molecules. In this case, lncRNAs respond to the environmental stimuli and then are transcribed at a specific time and space. This property makes them act as biomarkers for specific biological events. (2) They could act as decoy molecules by binding to the regulatory factors of transcription. For example, lncRNAs could bind to RNA-binding proteins, transcription factors, or chromatin modifiers to inhibit their biological activity. (3) They could function as guide molecules to direct the localization of regulatory factors. For example, lncRNAs can directly bind to protein molecules to form ribonucleoprotein complexes and mediate their precise localization to specific targets to regulate gene expression [19]. (4) lncRNAs could serve as scaffold molecules to assemble various effector molecules into macromolecules to achieve precise and specific control of biological events [19]. Finally, (5) lncRNAs could function as competing endogenous RNAs (ceRNAs) to sequester miRNAs, leading to the active transcription of their mRNA targets [20]. Several studies have shown that when TEs were embedded in lncRNAs, they may function in the processing, stability, and localization of lncRNAs. More importantly, TEs are often found to be the functional domains of lncRNAs [21]. For example, 73% of Linc-ROR sequences that have miRNA binding sites are derived from TE, and these sequences are essential for maintaining the pluripotency and self-renewal of embryonic stem cells [22]. Another example is XIST, which is important in early embryonic development and reproductive diseases [23]. XIST contains three functional repeat domains that are derived from TE. A-repeats that originated from ERVB5 TE are responsible for recruiting SPEN to silence the X chromosome; C-repeats, originating from ERVB4 TE, are required for the localization of XIST; and F-repeats, which are derived from a DNA transposon, are found to interact with JARID2 [24–28].

In mammals, development starts from the fusion of mature germ cells, sperms, and eggs, generating a totipotent zygote. Then, the zygote differentiates to form pluripotent stem cells that have the potential to give rise to an entire organism, including germ cells [29]. Thus, germ cells are the most remarkable cell type capable of reestablishing totipotency and transmitting heritable genetic and epigenetic information between generations [30]. Understanding the unique cell fate change from totipotent embryos to pluripotent stem cells and germ cells will enable us to develop novel strategies for disease treatments, particularly in regenerative medicine [31]. Although substantial progress has been made to dissect the molecular mechanism underpinning this cell fate change, the role of lncRNAs remains largely unknown. In this article, we have reviewed the recent progress of lncRNAs studies in embryogenesis, pluripotency, and reproduction, aiming to shed light on future research to probe the genetic program that drives the multistep developmental processes.

## 2. lncRNAs in Early Human Embryonic Development

lncRNAs are present from the beginning of human embryo development. After embryonic gene activation (EGA), lncRNAs become the main category of transcripts [14]. RNA-seq and hierarchical clustering analysis demonstrated that lncRNAs show distinct developmental stage-specific expression patterns [32]. Furthermore, the epigenetic signatures of lncRNAs are similar to those of protein-coding genes, including methylation distribution at the transcription start site (TSS), methylation dynamics, and negative correlation between gene expression and promoter methylation level. Collectively, these data suggest that lncRNAs may play essential roles in early human embryonic development by regulating gene expression [33].

Human endogenous retroviruses (HERV) are remnants from ancient germline infections by exogenous retroviruses and account for 8% of the human genome [34]. HERV-derived lncRNAs are found to express at specific stages and function in human-specific or even individual-specific aspects of early human embryo development [35]. HERVK is activated by the master transcription regulator of pluripotency, OCT4, from embryonic genome activation at the eight-cell stage to human embryonic stem cell derivation. It is involved in the immunoprotective process of human embryos against exogenous viral infection [36]. Another species of HERV, HERVH, is considered the most successful endogenous retrovirus in the human genome. It is expressed during human preimplantation embryogenesis and regulates human pluripotency by providing alternative binding sites for key transcription factors, functioning as a long-range enhancer, and producing pluripotency-specific lncRNAs [37].

Human pluripotency-associated transcripts 2, 3, and 5 (HPAT2, HPAT3, and HPAT5) are derived from transposable elements (TEs) and are essential for preimplantation embryo development by modulating the acquisition of pluripotency and the formation of the inner cell mass [38].

In addition, the activity of the X chromosome is regulated by the antagonistic action of lncRNAs XIST and XACT in the early development of human embryogenesis [39].

## 3. lncRNAs in Pluripotent Stem Cells

**Figure 3:**
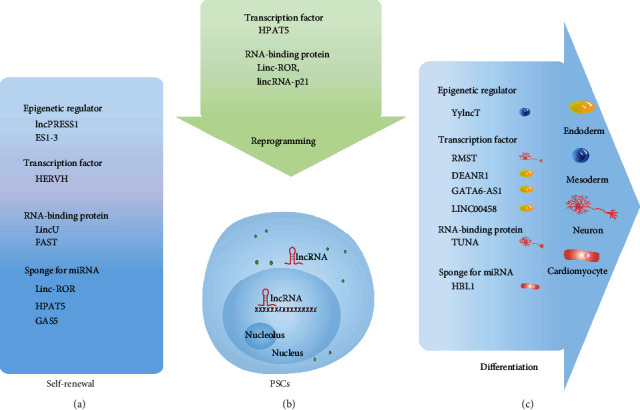
Mechanisms of lncRNAs in pluripotency, differentiation, and reprogramming of PSCs by interacting with different partners. Epigenetic regulator: recruit chromatin modification factors to affect chromatin status; transcription factor: binding transcription factors to regulate gene transcriptional activity; RNA-binding protein: interacting with RNA-binding protein to directly regulate protein activity; sponge for miRNA: functioning as the sponge of endogenous miRNA, preventing miRNA targets from degradation.

Pluripotent stem cells (PSCs) cultured *in vitro* provide a unique model for studying the molecular mechanisms of human embryogenesis [40] and are considered the seed cells to differentiate into functional cells for cellular therapeutics [41]. The core regulatory network for self-renewal and pluripotency involves transcription factors, chromatin modifiers, and lncRNAs [42, 43](Figure 3). PSCs express a characteristic set of lncRNAs that interact with the other members of the core regulatory network to (1) regulate gene expression, (2) modulate signaling pathways, (3) maintain epigenetic signatures, and (4) direct differentiation.

Linc-RoR, HERVH (human endogenous retrovirus subfamily H), HPAT5, and GAS5 (growth arrest-specific transcript 5) are found to be preferentially expressed in PSCs and interact with the core regulatory transcription factor network (OCT4, NANOG, SOX2, and SALL4) to regulate the gene expression profiles and safeguard pluripotency [22, 38, 44, 45]. Mechanically, Linc-RoR works as a competing endogenous RNA to connect the network of miRNAs with core transcription factors in PSCs. Linc-ROR prevents the core transcription factors from miRNA-mediated suppression in PSCs, thus regulating the self-renewal and pluripotency of PSCs [22]. HPAT5 acts as a miRNA sponge to modulate the balance between pluripotency and differentiation by counteracting the activity of let-7 [38].

Another group of lncRNAs, such as LincU, FAST, and GAS5, maintains the pluripotency of PSCs by modulating signaling pathways that are essential for PSCs [45–47]. Mechanistically, LincU binds to DUSP9 protein, an ERK-specific phosphatase, and stabilizes its expression, thereby inhibiting the MAPK/ERK signal pathway and maintaining the naive state of ESCs [46].

Examples of lncRNAs that modulate the epigenetic status of PSCs include ES1-3 and IncPRESS1. They are shown to function as molecular scaffolds that bridge different chromatin modifiers to maintain the epigenetic signatures of PSCs. ES1-3 are highly expressed in undifferentiated hESCs. As a modular scaffold, they recruit the suppressive PRC2 component SUZ12 to silence the SOX2 neural targets in PSCs, thus maintaining pluripotency [48–50].

lncRNAs are also involved in the differentiation of PSCs into three germ layers. RMST and TUNA (Tcl1 upstream neuron-associated lincRNA) promote neuronal differentiation of human PSCs [48, 49], while DEANR1, GATA6-AS1, and LINC00458 promote endodermal lineage specification [51–53]. For example, RMST interacts with SOX2 and binds to the promoter regions of neurogenic target genes to promote neuronal differentiation [48, 49]. DEANR1, an endoderm-specific lncRNA, interacts with SMAD2/3 to activate the expression of FOXA2, thus enabling the differentiation towards endoderm [51]. In addition, HBL1, BANCR, and YyIncT are identified as critical regulators for mesoderm development [54–56].

lncRNAs are also involved in reprogramming. Linc-ROR, as a negative regulator of p53, directly binds to heterogeneous nuclear ribonucleoprotein I (hnRNP I) to inhibit the expression of p53, thereby inhibiting p53-mediated cell cycle arrest and apoptosis and promoting cell reprogramming [57]. HERVH is significantly upregulated in the reprogramming process of fibroblasts to induce pluripotent stem cells (iPSCs). By recruiting P300 and OCT4 to the HERVH LTR7 region, HERVH regulates the expression of neighboring genes, as well as pluripotency-associated transcripts. It is suggested that HERVH plays an essential role in the acquisition of somatic pluripotency [44]. lincRNA-p21 (P53-induced large intergenic noncoding RNA p21) interacts with the H3K9 methyltransferase SETDB1 and the DNA methyltransferase DNMT1 through the RNA-binding protein HNRNPK to maintain high levels of H3K9me3 modification and/or CpG methylation at the pluripotency gene promoter, thus hindering somatic cell reprogramming [58]. Knockdown of HPAT5 impairs reprogramming, indicating that it contributes directly to reprogramming and acquisition of pluripotency [38].

## 4. lncRNAs in Human Germ Cell Development

Germ cell development is a complex differentiation process essential for the generation of gametes, which pass on the genetic information between generations [59]. Disruption of germ cell development or misregulation of gene expression in germline-related cells leads to infertility or reproductive diseases [60]. This dynamic developmental process is precisely regulated by a tissue- or cell-specific gene network [61]. As a new regulator in gene expression networks, cell type-specific lncRNAs have recently been discovered and suggested to be involved in many cellular processes during human germ cell development [62]. Several lncRNAs show differential expression or regulatory roles in the development of human primordial germ cells (hPGCs), the first progenitor cells of the germline [63]. For example, HIPSTR (heterogeneously expressed from the Intronic Plus Strand of the TFAP2A-locus RNA) has been identified as a novel lncRNA transcribed from the TFAP2A locus and shows differential expression in human primordial germ cells [64]. In addition, XACT and XIST are expressed to regulate X-chromosome dosage in hPGCs before meiosis [65]. RNA-seq analysis of human testicular cells has identified thousands of syntenic lncRNAs associated with spermatogenesis [66–71]. The narcolepsy candidate-region 1 gene (NLC1-C), a lncRNA expressed in the cytoplasm of spermatogonia and early spermatocytes, is found to be associated with male infertility and promotes testicular embryonal carcinoma cell proliferation [71]. Single-cell RNA-seq profiling of metaphase II oocytes also found 8,700 maternal lncRNAs expressed in the preimplantation embryos [32]. Note that a large number of RNA-binding proteins are found to be critical for germ cell development across species, including VASA (DDX4) and DAZL (Deleted in Azoospermia Like) [72]. These proteins might function by influencing lncRNA action to reinforce germ cell fate.

## 5. lncRNAs in Reproductive Diseases

Besides the roles in development, differential expression of many lncRNAs has been identified using microarray or RNA-seq between control and reproductive disease samples [73], indicating potential roles in pathogenesis. Although most of their functions and mechanisms of action need to be further annotated and characterized, these lncRNAs could serve as potential targets for the diagnosis and treatment [74] (Table 1).

### 5.1. lncRNAs Associated with Male Infertility

Spermatogenesis is a complex developmental process that is essential for male fertility [75]. The process is classified into three major phases: (1) mitotic proliferation of spermatogonia, (2) the meiosis of spermatocytes, and (3) spermiogenesis and maturation of spermatocytes to spermatozoa [76]. Each phase is strictly regulated by transcriptional factors, hormones, epigenetic regulators, and lncRNAs. Disruption of any steps of spermatogenesis, referred to as maturation arrest (MA), causes male infertility [77]. Nonobstructive azoospermia (NOA) is considered the most severe case of male infertility, and it is characterized as no sperm in the ejaculate due to failure of spermatogenesis [78]. Several lncRNAs have been indicated to play roles in the process of spermatogenesis and NOA.

The *narcolepsy candidate-region 1 gene* (*NLC1-C*, *also known as LINC00162*) is expressed in spermatogonia and primary spermatocytes. Compared with fertile controls, its expression is significantly downregulated in the cytoplasm and accumulated in the nucleus in the testis of infertile MA patients. NLC1-C forms a regulatory feedback loop with miR-320a and miR-383 to control the survival and proliferation of the germ cells in the process of spermatogenesis. In the cytoplasm, NLC1-C is the target of miR-320a and miR-383; while accumulated in the nucleus of spermatogonia and primary spermatocytes, it is suggested to repress the expression of miR-320a and miR-383 by direct binding to nucleolin, resulting in the hyperactive proliferation of germ cells, which leads to male infertility [71].

*GM2044* is indicated to play an essential role in NOA and specific in reproductive diseases. It is the miR-202 host gene, and its expression is significantly increased with its host gene miR202 in NOA of spermatogonial arrest. lncRNA Gm2044 inhibits the proliferation of the human testicular embryonic carcinoma cell NCCIT through the miR-202-Rbfox2 molecular signal pathway [79].

The expression of *Hox transcript antisense intergenic RNA* (*HOTAIR*) is decreased in asthenozoospermic and oligoasthenozoospermic patients [80]. The low expression of HOTAIR was also observed to be associated with specific sperm function parameters, including motility and vitality. It is found that low HOTAIR leads to downregulation of nuclear factor erythroid 2-related factor 2 (NRF2), a gene related to the expression of antioxidant genes and the quality of spermatozoa [81]. This eventually results in reactive oxygen species- (ROS-) related defects in sperm function.

lncRNA growth-arrested DNA damage-inducible gene 7 (*Gadd7*) is indicated in the regulation of the oxidative stress response and specific in reproductive diseases. Its expression is upregulated in patients with varicocele compared with fertile controls. Further functional analysis in mouse cell lines indicates that overexpression of gadd7 inhibits cell growth and promotes apoptosis by upregulating the proapoptotic regulator Bax and downregulating the antiapoptotic regulator Bcl2, resulting in male infertility [82].

### 5.2. lncRNAs Associated with Prostate Tumors

Prostate cancer is the most common cancer among men, and the androgen receptor (AR) plays a central role in its progression by regulating the expression of genes associated with the identity and behavior of prostate cancer cells [83]. A number of lncRNAs are identified as potential regulators for disease progression and may be applied as novel therapeutic targets.

*PRNCR1* and *PCGEM1* are highly expressed in aggressive prostate cancer and bind to AR successively. They enhance the activation of ligand-dependent and ligand-independent AR-mediated genes and promote the proliferation of prostate cancer cells [84].

*Nuclear-rich transcriptase 1* (*NEAT1*), a potential target of estrogen receptor alpha (ER*α*), is significantly overexpressed in prostate cancer. NEAT1 is shown to regulate the expression of prostate cancer genes and promotes the development of prostate cancer by changing the epigenetic landscape of the target gene promoter [85].

*PCAT-1* is upregulated in prostate cancer and promotes the proliferation of prostate cancer cells through PRC2 and cMyc proteins [86].

*MALAT-1* is upregulated in prostate cancer and is associated with the increase in the Gleason score, prostate-specific antigen (PSA), and tumor stage. Downregulating the expression of MALAT-1 inhibits the migration, invasion, and growth of prostate cancer cells, increases the rate of apoptosis, and blocks the cell cycle [87].

*SChLAP1* is highly expressed in prostate cancer and is associated with a poor prognosis. Thus, it could be used as an essential biomarker to identify patients with a high risk of lethal prostate cancer [88].

*GAS5* is downregulated in prostate cancer cells compared with prostate epithelial cells. GAS5 inhibits prostate cancer cell proliferation. It can bind directly to E2F1 and activate the P27^Kip1^ which is a regulator of the cell cycle. Thus, GAS5 induces a cell cycle arrest in the G0–G1 phase and acts as a tumor suppressor [89].

### 5.3. lncRNAs Associated with Ovarian Cancer

Ovarian cancer is one of the most common gynecological cancers that affect women's health worldwide. As there has been no effective method to detect ovarian cancer at an early stage, most patients are diagnosed in an advanced stage, which has developed resistance to multiple treatment modalities [90]. Despite the revolutionary role of surgery and chemotherapy in curing ovarian cancer, the overall prognosis of ovarian cancer is poor. Thus, improving our understanding of the pathogenesis of ovarian cancer is essential for developing more effective treatments.

*XIST* encodes a specific spliced lncRNA, and it is a vital regulator of X chromosome inactivation. It is identified to be the most differentially expressed gene and downregulated in recurrent ovarian tumors. Downregulation of Xist may increase the expression of linked inhibitors of apoptosis protein (X-linked Inhibitor of Apoptosis Protein (XIAP)) and lead to the phenotype of drug resistance [91].

*H19* is significantly increased in ovarian cancer cells and ovarian cancer tissues. Ectopic expression of H19 promotes cell proliferation while silencing the expression of H19 by RNA interference inhibits the growth of ovarian cancer cells and induces cell cycle arrest and apoptosis [92]. Moreover, overexpression of H19 enhances the ability of tumor cells to invade *in vitro* and metastasize *in vivo* [93].

*Metastasis-associated lung adenocarcinoma transcript 1* (*MALAT1*) is one of the earliest cancer-related lncRNAs identified to be related to ovarian cancer [94]. The expression level of MALAT1 is associated with ovarian cancer cells with different metastatic potentials. MALAT1 may play a role in the metastasis of epithelial ovarian cancer cells, but its mechanism needs to be further studied [95]. Knockdown of MALAT1 in ovarian cancer cells changes the expression of many genes related to cell proliferation, metastasis, and apoptosis, and inhibition of MALAT1 can significantly inhibit the tumorigenicity of SKOV3 cells [96].

*LINC00565* is highly expressed in ovarian cancer tissues, and its expression level was negatively correlated with the prognosis of patients with ovarian cancer. It has been found that the expression level of LINC00565 is related to the FIGO (International Federation of Gynecology and Obstetrics) stage and the size of tumor cells. Knockdown of LINC00565 in ovarian cancer cells inhibits the proliferation, invasion, and migration of the cells and induces cell cycle arrest. *In vivo* studies have shown that downregulating the expression of LINC00565 has an inhibitory effect on the growth of ovarian cancer cells by mediating the expression of cell cycle-related genes [97].

*DARS-AS1* is expressed higher in ovarian cancer tissues than in adjacent normal tissues. It promotes the migration and invasion of ovarian cancer cells. MicroRNA-532-3p (miR-532-3p) is identified as the direct target of DARS-AS1 in ovarian cancer, and DARS-AS1 via sponging miR-532-3p promotes the proliferation, migration, and invasion of ovarian cancer cells [98].

*FEZF1-AS1* is identified as a carcinogenic gene in ovarian cancer, as it is highly expressed in ovarian cancer tissues compared with adjacent normal tissues. Its expression is associated with a poor prognosis. After knocking down FEZF1-AS1, the proliferation of ovarian cancer cells was inhibited, and apoptosis was promoted. The mechanistic analysis found that FEZF1-AS1 regulated the JAK-STAT3 signal pathway by regulating the phosphorylation of STAT3 [99].

*LEF1-AS1* is upregulated in ovarian cancer and is related to poor prognosis. The absence of LEF1-AS1 results in the inhibition of proliferation, migration, and invasion of ovarian cancer cells. LEF1-AS1 interacts with miR-1285-3p, a tumor suppressor in ovarian cancer, to inhibit the expression of miR-1285-3p and promote the growth and metastasis of ovarian cancer cells [100].

### 5.4. lncRNAs Associated with Endometrial Carcinoma (EC)

Endometrial carcinoma is the most common cancer in the uterus. It is formed by the outgrowth of the cells that develop the glands in the endometrium. Although it tends to have a favorable prognosis if an early sign of abnormal uterine bleeding is presented, once it develops into metastasis or recurrence, the patients are at a significantly higher risk of mortality, with a median overall survival time of <16 weeks [101]. The genetic factors that cause endometrial carcinoma remain unclear, and a growing number of studies have associated lncRNAs with its initiation and progression.

*H19* is expressed higher in EC and tumor tissues than in the normal endometrial epithelium, and it regulates migration and invasion of the tumor cells [102].

*Colon cancer-associated transcript 1* (*CCAT1*) is expressed significantly higher in EC and tumor tissues than in normal endometrial tissue. Downregulation of CCAT1 expression leads to the inhibition of tumor cell growth and metastasis. In addition, it was found that CCAT1 was the direct target of miR181a-5p in endometrial carcinoma cells. It promotes the proliferation and migration of endometrial cancer cells by negatively regulating the expression of miR-181a-5p [103].

*MIR22HG* has been identified as a tumor repressor in EC. Its expression is significantly downregulated in endometrial carcinoma tissue. Functional tests *in vitro* showed that increased expression of MIR22HG could inhibit the proliferation and promote the apoptosis of cancer cells. In addition, the study proposed that MIR22HG inhibits the proliferation and migration of cancer cells by regulating the miR-141-3p/DAPK1 axis [104].

*Maternal expression gene 3* (*MEG3*) is a tumor suppressor gene, and its expression level in EC tissue is significantly lower than that in normal endometrial tissue. High expression of MEG3 inhibits the migration, invasion, and proliferation of EC cells and increases apoptosis, probably through the PI3K/mTOR signal transduction pathway [105].

### 5.5. lncRNAs Associated with Endometriosis

Endometriosis is a benign gynecological disorder characterized by the presence of endometrial cells from the lining of the uterus outside of the uterine cavity. Although research efforts have been devoted to uncovering the underlying cause of endometriosis, the pathophysiological mechanisms causing this disease remained obscure. Recent studies, especially the results from high-throughput RNA sequencing [106], have shown differential expression of lncRNAs in endometriosis-related tissues and indicate the contribution of lncRNAs to the pathogenesis of endometriosis.

*AC002454.1* is upregulated with cyclin-dependent kinase-6 (CDK6) in patients with endometriosis, and there was a significant positive correlation between them. After downregulating the expression of AC002454.1 and CDK6, the ability of cells to migrate, invade, and proliferate decreased, the proportion of cells in the S phase decreased, and the proportion of cells in the G0/G1 phase increased. Therefore, AC002454.1 and CDK6 have a synergistic effect on the biological behavior of endometrial cells [107].

*MALAT1* plays a vital role in endometriosis. Compared with normal tissues, the expression of MALAT1 in endometriosis is upregulated. Knockdown of MALAT1 inhibits the proliferation and migration of endometrial cells, enhances the activity of caspase-3, and induces apoptosis by inhibiting the NF-*κ*B/iNOS signal pathway [108].

*AFAP1-AS1* is significantly upregulated in ectopic endometrial tissues and is positively correlated with epithelial-mesenchymal transition (EMT). Knocking down AFAP1-AS1 can inhibit the activity of the EMT-related transcription factor ZEB1, thus inhibiting the EMT process of endometriosis [109].

*CCDC144NL-AS1* is a newly identified lncRNA whose expression is upregulated in ectopic endometrium tissues. Downregulation of CCDC144NL-AS1 inhibited the migration and invasion of EC cell lines. Mechanism studies have shown that the knockdown of CCDC144NL-AS1 leads to changes in the distribution of filamentous actin (F-actin) stress fibers in the cytoskeleton and affects the cytoskeleton structure. In addition, the expression of the CCDC144NL-AS1 gene promotes the protein expression of vimentin filament and matrix metalloproteinase-9 (MMP-9), which promotes cell invasion and migration [110].

### 5.6. lncRNAs Associated with Cervical Cancer

Cervical cancer is one of the most frequently diagnosed malignant gynecological cancers that endanger women's health and lives [111]. Increasing data have shown the regulatory roles of lncRNAs in the pathogenesis of cervical cancer, with the prospective clinical application in the diagnosis and treatment of cervical cancers.

In cervical cancer, the expression of IGF2 was significantly increased, and the expression of *H19* was decreased considerably. However, the mechanism of this disorder is not precise, and further research is needed [102].

*MALAT1* is identified as an essential regulatory factor involved in the occurrence of cervical cancer. Its expression in cervical cancer tissues is significantly higher than that in normal tissues. When endogenous MALAT1 is knocked out, it reduces the proliferation and invasion of cervical cancer cells and promotes apoptosis [112].

The expression of *HOTAIR* in cervical cancer is higher than that in normal tissues. HOTAIR has indicated a role in metastasis and invasion of tumor cells by regulating the expression of vascular endothelial growth factor, matrix metalloprotein-9, and epithelial-to-mesenchymal transformation- (EMT-) related genes [113].

The expression level of *RP11-480I12.5* in the cervical carcinoma cell line is higher than that in normal tissue. RP11-480I12.5 induces EMT through the Wnt/*β*-catenin pathway and promotes cervical cancer cell lines' migration, invasion, and proliferation [114].

*lncRNARP1-93H18.6* is expressed higher in paracancerous tissues in cervical cancer and specific in cervical cancer. Overexpression of RP1-93H18.6 promotes growth and metastasis of tumor cells and reduces apoptosis. Knocking down the expression of lncRNARP1-93H18.6 promotes apoptosis and inhibits the development of cervical carcinoma cells by blocking the PI3K/Akt/mTOR pathway [115].

*DSCAM-AS1* is related to the occurrence and development of various tumors, and its role in cervical cancer has recently been studied. The expression of DSCAM-AS1 in cervical carcinoma is increased. DSCAM-AS1 enhances the ability of cells to migrate, invade, and proliferate and promotes the development of cervical cancer through regulating the miR-877-5p/ATXN7L3 axis [116].

GAS5 is a tumor suppressor factor that inhibits proliferation, EMT, invasion, and metastasis of tumor cells. *GAS5-AS1* is the antisense RNA of GAS5, located on chromosome 1q25.1. Compared with normal tissues adjacent to cancer, the expression of GAS5-AS1 in cervical cancer is downregulated, and its expression is related to the FLGO stage, lymphatic metastasis, distant metastasis, and poor prognosis in patients with cervical cancer. Mechanistically, GAS5-AS1 regulates the tumor suppressor GAS5 in an ALKBH5-m6A-YTHDF2-dependent manner. Specifically, GAS5-AS1 reduced the level of GAS5N6-methyladenosine (M6A) modification and improved the stability of GAS5 through the interaction of RNA demethylase and ALKBH5. In addition, YTHDF2 specifically recognizes and binds to the RNA containing M6A and degrades M6A-modified transcript [117].

*Plasmacytoma variant translocation-1* (*PVT1*) promotes the proliferation and metastasis of cervical cancer. The expression of PVT1 is upregulated in cervical cancer cells, and PVT1 binds directly to miR-140-5p, which promotes the expression of Smad3 and then promotes the development of cervical cancer [118].

### 5.7. lncRNAs Associated with Polycystic Ovary Syndrome (PCOS)

Polycystic ovary syndrome (PCOS) is one of the most common metabolic and reproductive disorders that has been estimated to affect approximately 5 to 20% of reproductive-aged women worldwide [119]. Although the etiology of PCOS remains unclear, most researchers believe that the causes are multifactorial, and lncRNAs have recently been suggested to play pivotal roles in its pathogenesis and prognosis.

*H19* is suggested to be involved in the occurrence and development of PCOS. In patients with PCOS, the expression of H19 is increased. The expression level of fasting plasma glucose (FPG), a sensitive indicator in the early stage of metabolic disease, is positively correlated with H19 in PCOS patients. These results suggest that the expression of H19 may be a critical factor in endocrine and metabolic disorders in patients with PCOS [120].

Taken together, many lncRNAs, including H19, NEAT1, MALAT1, HOTAIR, and PVT1, are upregulated in the progression of many reproductive diseases. Interestingly, the expression of several lncRNAs, which is highly expressed in embryonic development, is reactivated in the development of reproductive cancer. For example, H19 is highly expressed in embryonic stem cells and essential for early human embryonic development. While its expression is downregulated after birth, the expression of H19 is significantly upregulated in endometrial carcinoma and ovarian cancer [121]. Recently, the reemergence of fetal-associated features in the tumor ecosystem is getting much attention and is referred to as oncofetal reprogramming [122]. Upregulation of specific lncRNAs in reproductive cancer development could be one of the features reminiscent of fetal development and serves as one of the potential targets for therapeutic interventions.

## 6. Conclusion and Future Perspectives

With the advances in sequencing technology, especially at the single-cell level, more and more lncRNAs have been identified at specific stages or within a particular type of cells, during human embryo and reproductive development. While expanding the repositories of lncRNAs, we notice that a unique subset of lncRNAs is expressed during human development. Dissection of the function of human-specific lncRNAs may be of preeminent importance for understanding the unique specifics of human development.

As a newly discovered role in gene regulatory networks, lncRNAs provide an additional layer of complexity for transcriptional and posttranscriptional regulation of gene expression programs. In addition, an increasing number of lncRNAs are differentially expressed within the disease tissues. They were found to regulate the initiation and progression of reproductive diseases through mediating the gene expression program. However, most of the functional results are based on the analysis *in vitro* on disease-related cell lines. Rigorous investigations *in vivo* or in organoids that resemble the physiological environment of development or diseases are necessary to reveal the biological and physiological functions of lncRNAs.

lncRNAs are proposed as therapeutic or diagnostic targets for disease treatment, as many of their expression are restricted to a specific tissue/or cell type within a specific cellular stage, which renders superior specificity. Furthermore, the diversity of strategies to target lncRNAs offers a wide range of therapeutic options. At the transcription level, we can inhibit the expression of lncRNAs by genome editing techniques or upregulate their expression by knockdown of the corresponding natural antisense transcripts (NATs). At the posttranslational level, lncRNAs can be degraded by nucleic acid-based approaches, including siRNAs, antisense oligonucleotides (ASO), and morpholinos.

Although immense enthusiasm is aroused in the field of lncRNA-based therapy, especially nucleic acid-based approaches, several challenges must be addressed before the progression to large-scale clinical applications. First, we need to have a thorough understanding of the molecular function of lncRNAs to identify disease-determining lncRNAs. Second, robust and physiologically relevant preclinical models need to be established. As we mentioned above, a few lncRNAs associated with diseases are human/primate-specific or even patient-specific. So patient-derived xenograft models or 3D organoids have gained much interest in preclinical research. Third, for nucleic acid-based therapies, a lack of an efficient delivery system to cross the cellular plasma membrane, the risk of the overactivating innate immune response, and the possibility of the off-target effect are the main issues that need to be solved.

## Figures and Tables

**Table 1:** lncRNAs and their functions in reproductive diseases.

Diseases	lncRNA	Full name	Expression level	Assessed cell line	Signaling pathways and molecules	Functions	In other diseases	References
Nonobstructive azoospermia (NOA)	NLC1-C	Narcolepsy candidate-region 1 gene	Downregulated	NCCIT, NTERA-2 (NT2), HEK293 T	Sponge for miR-320a, miR-383	Inhibits miR-320a and miR-383 transcripts by binding to nucleolin, resulting in a hyperactive proliferation of germ cells	Testicular embryonal carcinoma	[71]
	GM2044	—	Upregulated	NCCIT	miR-202-Rbfox2 pathway	Inhibits the proliferation of the human testicular embryonic carcinoma cell NCCIT	—	[79]
	HOTAIR	Hox transcript antisense intergenic RNA	Downregulated	—	NRF2	Relates to defects in sperm function	Breast cancer, lung cancer, and pancreatic cancer	[80, 81, 123]
	Gadd7	lncRNA growth-arrested DNA damage-inducible gene 7	Upregulated	GC-1, GC-2	Bax, Bcl2	Inhibits cell growth and promotes apoptosis by upregulating the proapoptotic regulator Bax and downregulating the antiapoptotic regulator Bcl2	—	[82]
Prostate tumors	PRNCR1/PCGEM1	Prostate cancer-associated noncoding RNA 1/PCGEM1 prostate-specific transcript	Upregulated	LNCaP, LNCaP-cds1, LNCaP-cds2, CWR22Rv1	AR	Promotes the proliferation of prostate cancer cells	Breast cancer and lung cancer	[84, 124]
	NEAT1	Nuclear-rich transcriptase 1	Upregulated	LNCaP and PC3, RWPE1, VCaP and DU145	Estrogen receptor alpha (ER*α*)	Promotes the development of prostate cancer	Non-small-cell lung cancer, breast cancer, and hepatocellular carcinoma	[85, 125]
	PCAT-1	Prostate cancer-associated transcript-1	Upregulated	LNCaP	PRC2, cMyc	Promotes the proliferation of prostate cancer cells	Colorectal cancer, hepatocellular cancer, and gastric cancer	[86, 126]
	MALAT-1	Metastasis-associated lung adenocarcinoma transcript 1	Upregulated	LNCaP-AI, 22RV1	ZEB1, ZEB2, Slug	Is associated with the increase in the Gleason score, prostate-specific antigen (PSA), and tumor stage and promotes the invasion and growth of prostate cancer cells	Glioma, hepatocellular carcinoma, and multiple myeloma	[87, 127]
	SChLAP1	Second chromosome locus associated with prostate-1	Upregulated	—	—	Relates to poor prognosis and could be used as an important biomarker to identify patients with a high risk of lethal prostate cancer	Triple negative breast cancer and bladder cancer	[88, 128, 129]
	GAS5	Growth arrest specific 5	Downregulated	PC3, DU145, and PNT2C2	E2F1, P27^Kip1^	Induces a cell cycle arrest in the G0–G1 phase and acts as a tumor suppressor	Colorectal cancer, gastric cancer, and melanoma	[89] [89, 130]
Ovarian cancer	XIST	Inactive X chromosome-specific transcripts	Downregulated	ALST, CAOV3, OVCA3, OVCA420, OVCA429, OVCA432, OVCA433, OVCA633, OVCA680, OVCA702, OVCA810, SKOV3, ES-2, TOV21G	XIAP	Downregulation of Xist may increase the expression of linked inhibitors of apoptosis protein and lead to the phenotype of drug	Non-small-cell lung cancer, breast cancer, and primary hepatocellular carcinoma	[91, 131, 132]
	H19	Imprinted maternally expressed transcript	Upregulated	SKOV3, OV90, TOV112D, ES2	Caspase-3, caspase-9, Bax, Bcl-2, cyclin B1/Cdc2	Promotes ovarian cancer cell proliferation	Head and neck cancer, pancreatic cancer, and osteosarcoma	[92, 93, 133]
	MALAT1	Metastasis-associated lung adenocarcinoma transcript 1	Upregulated	SKOV3, SKOV3.ip1, 293T	—	Promotes cell proliferation and metastasis and inhibits cell apoptosis	Glioma, hepatocellular carcinoma, and multiple myeloma	[94–96, 127]
	LINC00565	Long intergenic nonprotein coding RNA 565	Upregulated	OVCAR3, SKOV3, HO8910, A2780, and HEY	GAS6, cyclinE1, cyclinD1, CDK4 P16, P21	Relates to the FIGO (International Federation of Gynecology and Obstetrics) stage, cell cycle, and size of tumor cells and promotes cell proliferation, invasion, and migration	Gastric cancer and colorectal cancer	[97, 134, 135]
	DARS-AS1	DARS1 antisense RNA 1	Upregulated	A2780, SKOV3, and OVCAR-3	Sponge for miR-532-3p	Promotes the proliferation, migration, and invasion of ovarian cancer cells	Thyroid cancer, clear cell renal cell carcinoma, and non-small-cell lung cancer	[98, 136, 137]
	FEZF1-AS1	FEZF1 antisense RNA 1	Upregulated	SKOV-3, HO8910, HO8910PM, ES2, and HG-SOC	JAK-STAT3 pathway	Relates to poor prognosis, promotes cell proliferation, and inhibits cell apoptosis	Colorectal cancer, gastric neoplasia, and hepatocellular carcinoma	[99, 138]
	LEF1-AS1	LEF1 antisense RNA 1	Upregulated	SKOV3, OVCAR3	miR-1285-3p	The absence of LEF1-AS1 results in inhibiting proliferation, migration, and invasion of ovarian cancer cells	Glioblastoma, colorectal cancer, and retinoblastoma	[100, 139–141]
Endometrial carcinoma (EC)	H19	Imprinted maternally expressed transcript	Upregulated	—	—	Regulates migration and invasion of the tumor cells	Head and neck cancer, pancreatic cancer, and osteosarcoma	[102, 133]
	CCAT1	Colon cancer-associated transcript 1	Upregulated	HEC-1-A, KLE, Ishikawa	Sponge for miR-181a-5p	Promotes the proliferation and migration of endometrial cancer cells	Breast cancer and multiple myeloma	[103, 142]
	MIR22HG	MIR22 host gene	Downregulated	HEC-1 A, KLE	Sponge for miR-141-3p	Inhibits the proliferation and migration and promotes the apoptosis of cancer cells	Esophageal cancer, lung cancer, and hepatocellular carcinoma	[104, 143]
	MEG3	Maternal expression gene 3	Downregulated	Ishikawa, HEC-1B	PI3K/m-TOR pathway, BclxL, VEGFA	High expression of MEG3 inhibits the migration, invasion, and proliferation of EC cells and increases apoptosis	Gastric cancer, osteosarcoma, and breast cancer	[105, 144]
Endometriosis	AC002454.1	—	Upregulated	—	CDK6	Promotes the migration, invasion, and proliferation of cells and regulates the cell cycle	Bladder cancer	[107, 145]
	MALAT1	Metastasis-associated lung adenocarcinoma transcript 1	Upregulated	—	NF-*κ*B/iNOS pathway, MMP-9, caspase-3	Promotes the proliferation and migration of endometrial cells	Glioma, hepatocellular carcinoma, and multiple myeloma	[108, 127]
	AFAP1-AS1	Actin filament-associated protein 1 Antisense RNA1	Upregulated	Ishikawa	ZEB1	Promotes the EMT process of endometriosis	Esophageal cancer, pancreatic ductal adenocarcinoma	[109, 146]
	CCDC144NL-AS1	CCDC144NL antisense RNA 1	Upregulated	hEM15A	MMP-9, F-actin, vimentin	Affects the cytoskeleton structure and promotes cell invasion and migration	Osteosarcoma, gastric cancer, non-small-cell lung cancer, and hepatocellular carcinoma	[110, 147–150]
	TC0101441	—	Upregulated	ECSCs	TCF8/ZEB1, slug, snail, and N-cadherin	EV shuttling of TC0101441 promotes invasion and migration of endometriosis	Gastric cancer	[151, 152]
	UCA1	Urothelial carcinoma-associated-1	Downregulated	—	—	Is involved in the pathogenesis of endometriosis and can be used as a biomarker for diagnosis and prognosis	Urothelial carcinoma-associated 1 gastric cancer and colorectal cancer	[153, 154]
	H19	Imprinted maternally expressed transcript	Downregulated	293T, HESCs	H19/Let-7/IGF1R, H19/miR-216a-5p/ACTA2 pathway	Regulates endometrial stromal cell proliferation, invasion, and migration	Head and neck cancer, pancreatic cancer, and osteosarcoma	[133, 155, 156]
	aHIF	Antisense hypoxia-inducible factor	Upregulated	ECSCs, HUVECs	(VEGF)-A, VEGF-D	Facilitates endometriosis angiogenesis and is used as a potential biomarker and therapeutic target for endometriosis	Gastric cancer, glioblastoma multiforme, and paraganglioma	[157–159]
Cervical cancer	MALAT1	Metastasis-associated lung adenocarcinoma transcript 1	Upregulated	—	HeLa, CaSki	Promotes the proliferation and invasion of cervical cancer cells and reduces apoptosis	Glioma, hepatocellular carcinoma, and multiple myeloma	[112, 127]
	HOTAIR	Hox transcript antisense intergenic RNA	Upregulated	SiHa, HeLa, Caski	VEGF, MMP-9, E-cadherin, *β*-catenin, vimentin, snail, twist	Promotes metastasis and invasion of tumor cells	Breast cancer, lung cancer, and pancreatic cancer	[113, 123]
	RP11-480I12.5	—	Upregulated	PCS-480-011, SiHa (HTB-35), HeLa229 (CCL-2.1), and MS751	Wnt/*β*-catenin pathway	Induces EMT through the Wnt/*β*-catenin pathway and promotes migration, invasion, and proliferation of cervical cancer cell lines	Breast cancer	[114, 160, 161]
	RP1-93H18.6	—	Upregulated	SiHa, HeLa, CaSki, and C-33A	PI3K/Akt/mTOR pathway	Promotes growth and metastasis of tumor cells and reduces apoptosis	—	[115]
	DSCAM-AS1	DSCAM antisense RNA 1	Upregulated	SiHa, HeLa, C-33A, and CaSki	Sponge for miR-361-5p	Enhances the ability of cells to migrate, invade, and proliferate and promotes the development of cervical cancer	Non-small-cell lung cancer, colorectal cancer, and osteosarcoma	[116, 162]
	GAS5-AS1	GAS5 antisense RNA 1	Downregulated	Caski, SiHa, C33A, and HeLa	GAS5	Relates to the FLGO stage, lymphatic metastasis, distant metastasis, and poor prognosis and promotes proliferation, migration, and invasion	Glioma, non-small-cell lung cancer, and hepatocellular carcinoma	[117, 153, 163, 164]
	PVT1	Plasmacytoma variant translocation-1	Upregulated	HeLa and SiHa	Sponge for miR-140-5p, Smad3	Promotes the proliferation and metastasis of cervical cancer	Clear cell renal cell carcinoma and thyroid cancer	[118, 165]
Polycystic ovary syndrome (PCOS)	H19	Imprinted maternally expressed transcript	Upregulated	Peripheral blood leukocytes	FPG	May be a key factor in endocrine and metabolic diseases in patients with PCOS	Head and neck cancer, pancreatic cancer, and osteosarcoma	[120, 133]
	PVT1	Plasmacytoma variant translocation-1	Upregulated	—	Sponge for miR-17-5p	Regulates the apoptosis and the proliferation of ovarian granulosa cells	Clear cell renal cell carcinoma and thyroid cancer	[165, 166]
	LET	Low expression in tumor	Downregulated	KGN	Wnt/*β*-catenin and Notch pathways, TIMP2	Promotes cell migration and survival and inhibits cell apoptosis	Hepatocellular carcinoma, colorectal cancer, and squamous cell lung carcinoma tissues	[167, 168]
	TMPO-AS1	TMPO antisense RNA 1	Upregulated	COV434	Sponge for miR-355-5p	Serve as a potential target to treat PCOS	Lung cancer, breast cancer, and colorectal cancer	[169, 170]
	NEAT1	Nuclear-rich transcriptase 1	Upregulated	Ovarian tissue in rats	Sponge for miR-381, IGF1	Promotes cell proliferation and represses cell apoptosis	Non-small-cell lung cancer, breast cancer, and hepatocellular carcinoma	[125, 171]
	LINC00477	Long intergenic nonprotein coding RNA 477	Upregulated	Sponge for miR-128	Sponge for miR-128	LINC00477/miR-128 axis may represent a potential method for the treatment of PCOS	Gastric cancer	[172, 173]
